# Correction of failure in linear antenna arrays with greedy sparseness constrained optimization technique

**DOI:** 10.1371/journal.pone.0189240

**Published:** 2017-12-18

**Authors:** Shafqat Ullah Khan, M. K. A. Rahim, Murtala Aminu-Baba, N. A. Murad

**Affiliations:** 1 Advanced RF & Microwave Research Group, Department of Communication Engineering, Faculty of Electrical Engineering, Universiti Teknologi Malaysia, Skudai, Johor, Malaysia; 2 Department of Computer and Communications Engineering, Faculty of Engineering and Engineering Technology, Abubakar Tafawa University, Yelwa Bauchi, Nigeria; Beijing University of Posts and Telecommunications, CHINA

## Abstract

This paper proposes the correction of faulty sensors using a synthesis of the greedy sparse constrained optimization GSCO) technique. The failure of sensors can damage the radiation power pattern in terms of sidelobes and nulls. The synthesis problem can recover the wanted power pattern with reduced number of sensors into the background of greedy algorithm and solved with orthogonal matching pursuit (OMP) technique. Numerical simulation examples of linear arrays are offered to demonstrate the effectiveness of getting the wanted power pattern with a reduced number of antenna sensors which is compared with the available techniques in terms of sidelobes level and number of nulls.

## Introduction

This paper stresses on the problem of failure correction in linear antenna arrays which has many applications in satellite and radar communication systems [1–5]. The possibility of failure of one or more sensors in the communication system can damage the radiation power pattern in terms of sidelobes, nulls and the communication become a dream. To get the wanted power pattern with the active number of sensors is very important to continue the communication. The synthesis problem in antenna array is associated to find the weights and locations for the active sensors that produce a desired pattern. This technique focuses to get the wanted power pattern even in case of failure of antenna sensors. Detection and correction of faulty patterns in antenna arrays have received increasing attention in the recent years [6–22]. It is very important to detect the position of faulty sensors. Once the position of faulty sensors is detected [6–16], such as from a small number of far field measurements [6–8], detection on the basis of pattern [9], evolutionary algorithms [10–13] and compressed sensing techniques [14–16], then the correction techniques applied to recover the desired pattern. The pattern recovery techniques include evolutionary algorithms such as cuckoo search algorithm [17–18], cultural algorithm with differential evolution [19], genetic algorithm with pattern search [20], firefly algorithm [21], grey wolf optimizer along with interior point algorithm [22], particle swarm optimization for failed array compensation [23–24] and improved genetic algorithm [25]. For most of them, the pattern is recovered by adjusting the active sensors by only controlling the excitation weights of the antenna arrays. Such correction of array failure can simply be solved by the computational methods which is time consuming without reducing the number of sensors. In addition, most correction techniques in the literature deal only with the active sensors and requires a large computation to adjust the remaining sensors in the array to get the desired radiation power pattern. Another possibility to get the desired pattern is the time modulated linear arrays [26], conjugate gradient technique [27] and some analytical technique [28] achieved the sidelobes level only but can not resolve the issue of null placement and null depth level at the desired locations. Indeed, using nonuniform sensor spacings has more freedoms and can reduce the number of sensors to get the desired radiation power pattern. For the synthesis of a nonuniformly spaced array with single-pattern, many practical methods have been proposed, such as a convex optimization [29], sequential convex optimization [30] matrix pencil [31], extended matrix pencil algorithm [32] and sparseness optimization algorithms [33] are applied to get the desired pattern with reduced number of sensors. The failure correction of sensors by the greedy optimization algorithm is an interesting and efficient way to get the desired power pattern with the minimum number of sensors.

In this paper, the failure correction problem is developed from the greedy sparseness constrained optimization (GSCO) point of view. The objective is to develop the wanted pattern with the reduced number of sensors. The existing techniques use the minimum *L*_2_ optimization and resolve with global search optimization techniques which has mainly two problems. First, the global search optimization technique requires large computations and is time consuming, particularly for satellite communications. Secondly, the *L*_2_ norm minimization gives the approximate desired radiation power pattern, but does not guarantee with the reduced number of sensors. In this study, an antenna array failure correction problem is studied from the GSCO technique which finds as few non-zero values which correspond to the active sensors of the array in the recovered radiation power pattern. Suppose an array of *N* sensors with uniform spacing and some sensors in the array become damaged. The sensors which fail is corresponding to having no location in the antenna array. Therefore, we can say that the array sensor positions is sparse, so the active sensors is fewer than the total number of sensors in the the array antenna. The main aim of array failure correction is to get the desired pattern with minimum number of sensors whose weight excitation is not equal to zero. Thus the failure correction problem is ensemble as an optimization problem and solved by GSCO technique. The proposed solution provides better radiation pattern in terms of sidelobes and nulls than the existing methods with less number of sensors. The organization of the paper is planned as follows. Correction of linear arrays with greedy sparseness constrained optimization technique is offered In Section II. In section III, some simulation results is offered to confirm the effectiveness of the recovered pattern with the proposed solution. Some concluding remarks with future directions are discussed in Section IV.

### Linear array

Consider a non uniforrrm symmetrical linear array antenna consists of *N* number of sensors as shown in Fig 1. The healthy array factor for this setup is given by [34–35],

$$A\left(\theta \right)=\sum_{i=1}^{N}a_{i}e^{-jkd_{i}\cos\theta }$$ (1)

where *a*_*i*_ is the Chebyshev excitation weight of the *ith* sensor positioned at *d*_*i*_ and *k* is the wave number. It is supposed that the weight excitations of the antenna array are conjugate symmetrical. For an even *N* number of sensors, the weight excitations can be written as follows,

$${\left(a_{i}\right)}^{*}=a_{N+1-i}\;for\;n=1,2,\ldots .N/2$$ (2)

and for odd number of sensors it can be written as follows,

$$a_{N/2}=a_{N/2+1}$$ (3)

Through this condition, the array factor is a real valued and can be written as follows,

A(θ)=2Re{∑i=1Naie−jkdicosθ} (4)

Eq (2) can be written as

$$s\left(\theta \right)=\left[e^{-jkd_{1}\cos\theta },e^{-jkd_{2}\cos\theta },\ldots e^{-jkd_{N/2}\cos\theta }\right]$$

$$a_{i}={\left[a_{1},a_{2},\ldots a_{N/2}\right]}^{T}$$

A(θ)=2Re(s(θ)a) (5)

$$A\left(\theta \right)=\sum_{i=1}^{N/2}a_{i}\cos\left(kd_{i}\cos\theta \right)$$ (6)

Now if one or more sensors in the antenna array become damaged. The power pattern for this damage setup can be given by the following expression as follows

$$A_{d}\left(\theta \right)=\sum_{\begin{array}{l} i=1\\ i\neq l,m,n,q \end{array}}^{N}w_{i}e^{jkd_{i}\cos\theta }$$ (7)

It is supposed that the sensor *l* = *w*_11_,*m* = *w*_12_,*n* = *w*_13_,*q* = *w*_14_ becomes damaged in the antenna array. One can clearly monitor from Fig 2 that due to the sensor failure, the radiation power pattern disturbs in terms of sidelobes and nulls. The values of sidelobes level and null depth level of the initial and damaged array at different positions are given in Table 1. So, the main objective of this work is to correct the failure pattern with the minimum number of sensors that has the same desired pattern as the Chebyshev pattern. The proposed methodology is based on the greedy sparseness constrained optimization (GSCO) technique to correct the failure pattern with the minimum number of sensors.

**Fig 1:**
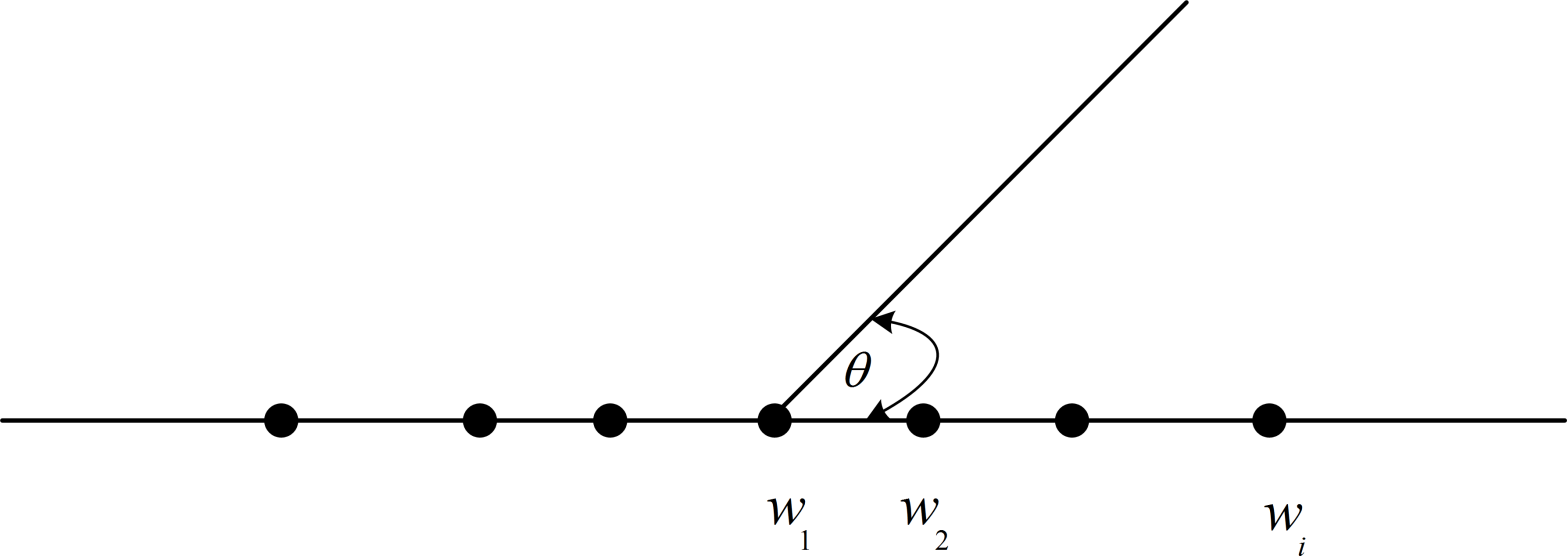
Non uniform symmetrical linear array.

**Fig 2:**
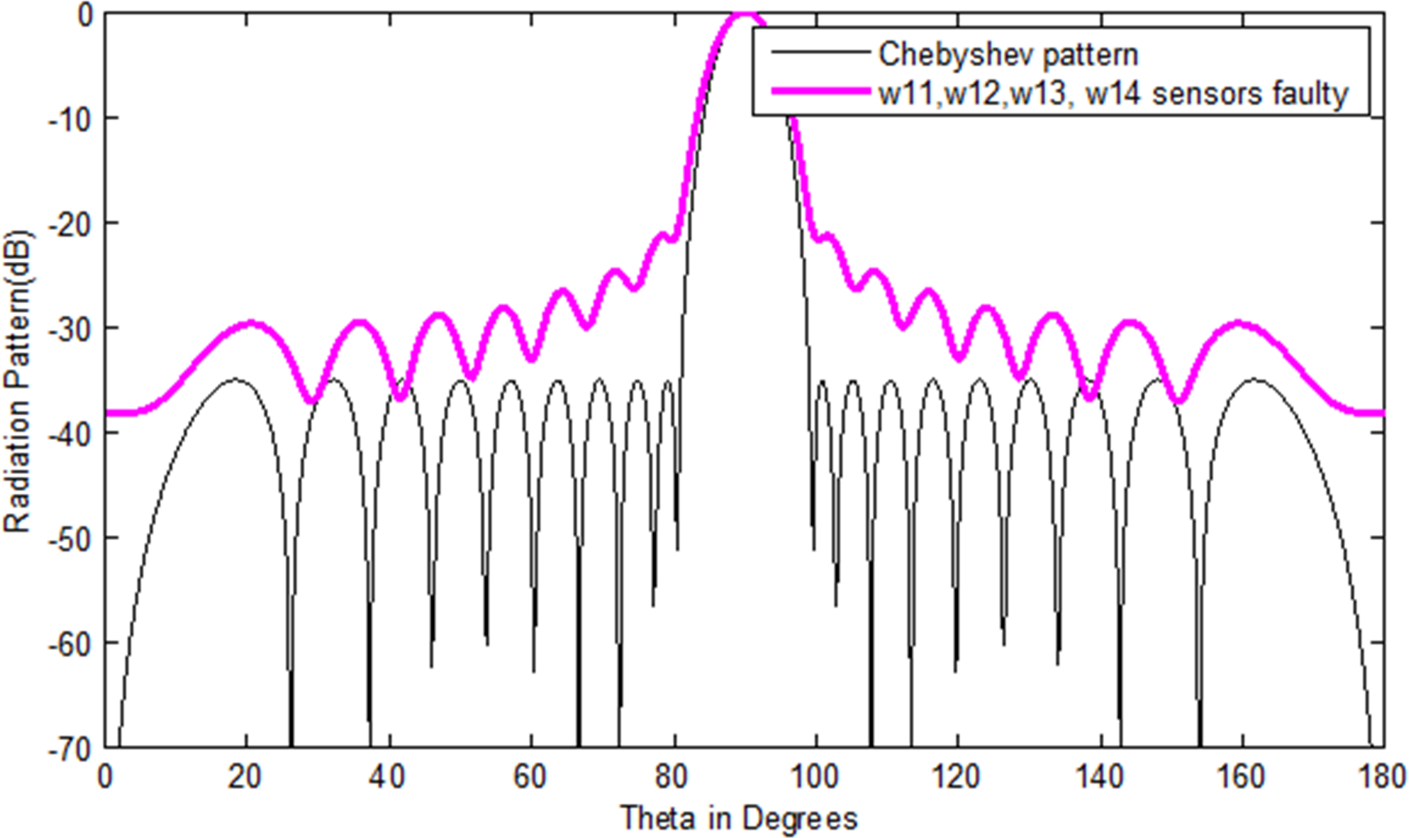
Chebyshev array pattern of 20 number of sensors with damaged sensor (*w*_11_,*w*_12_,*w*_13_,*w*_14_).

**Table 1:** Comparison analysis for initial and damaged array.

Initial array parameter			Damaged array parameter		
Null positions at an angle *θ*_*i*_	SLL (dB)	NDL (dB)	Null positions	SLL (dB)	NDL (dB)
					
2.1	-35.00	-70.00	-37.17	-29.69	-37.03
25.8	-35.00	-70.00	-36.83	-29.52	-36.48
36.9	-35.00	-70.00	-33.21	-28.87	-33.99
45.9	-35.00	-62.21	-32.86	-28.17	-32.63
53.7	-35.00	-60.35	-29.98	-26.62	-29.78
60.3	-35.00	-62.76	-26.32	-24.71	-26.31
66.9	-35.00	-70.00	-21.53	-21.29	-21.08
72.6	-35.00	-70.00			
77.1	-35.00	-56.57			

## Correction of array failure with greedy sparseness constrained optimization

This paper emphasis on the problem of correcting the failure pattern with reduced number of sensors in linear antenna array. The main objective is to get a recoverd array which has reduced number of sensors while keeping the desired power pattern as that of the original array. The cost function for the array failure correction can be defined as follows

$$Cost\;Fuction=const.^{\min\left(Q\right)}\left\{\underset{{\left\{w_{i},d_{i}\right\}}_{i=1,\ldots Q}}{\min}{\left\|A\left(\theta \right)-\sum_{i=1}^{Q}w_{i}e^{jkd_{i}\cos\theta }\right\|}_{2}\right\}$$ (8)

where *A*(*θ*) is the original Chebyshev pattern at different directions, *Q* is the minimum number of sensors of the recovered pattern, *w*_*i*_ is the excitation weight of the *ith* sensor of the recovered array at location *d*_*i*_ while *k* = 2*π*/*λ* is the wave number. The main goal is to recover the wanted pattern *A*(*θ*) with the reduced number of sensors under a cost function which gives minimum mean square error.

## Greedy sparseness constrained optimization technique

In this section, we develop the array failure solution based on greedy sparseness constrained optimization (GSCO) technique. As we had seen the failure of sensor, damage the radiation power pattern. The GSCO find as few non-zero values in a measurement matrix which represents the minimum number of sensors in the array. Suppose that the antenna sensors are placed symmetrically along the x-axis with uniform spacing. As we had assumed the failure of some sensors. Now there are two situations in the antenna array, one state radiating the waves while the other state is damaged which do not radiate. Eq (2) can be given in a matrix form as follows.

$$\left[A\right]={\left[M\right]}_{m\times n}{\left[w\right]}_{n\times 1}$$ (9)

$$\left[\begin{array}{l} A\left(\theta_{1}\right)\\ A\left(\theta_{2}\right)\\ \vdots \\ A\left(\theta_{m}\right) \end{array}\right]={\left[\begin{array}{l} M_{1,1}\;M_{1,2}\cdots M_{1,n}\\ M_{2,1}\;M_{2,2}\cdots M_{2,n}\\ \vdots \;\vdots \;\vdots \\ M_{m,1}\;M_{m,2}\cdots M_{m,n} \end{array}\right]}_{m\times n}{\left[\begin{array}{l} w_{1}\\ w_{2}\\ \vdots \\ w_{n} \end{array}\right]}_{n\times 1}$$ (10)

where the number of samples is of the power pattern is *m* and *n* is the reduced number of sensors required to get the wanted pattern. **A** is *m* × 1 vector having the radiation power pattern in different directions, **M** is *m* × *n* measurement matrix of steering vectors having *m* < *n* and **w** is the *n* × 1 excitation weights of the minimum number of sensors required to get the desired pattern. Subsequently *w* has *m* nonzero values, the radiation vector **A** = **Mw** is a linear combination of *m* columns from **M**. To recover the desired pattern, we want to find that which columns of **M** contribute in the radiation vector **A**. In this technique the columns are picked in a greedy way. At every iteration, the colum of **M** are choosen that is intensely correlated with the radiation vector **A**. Then deduct off its impact to **A** and repeat on residual and after m repetitions the proposed technique will recoverd the desired pattern.

***Greedy OMP algorithm***

***Input***

        •          *m* × *n Measurement steering matrix* **M**

        •          *m* × 1 *Radiation vector* **A**

        •          **w** *is excitation weights of the recovered pattern*

***Output***

        •          $\hat{w}$ *is an estimate of the recovered pattern*

        •          **a**_*m*_ *of the radiation vector* **A**

        •          *Residual* **r**_*m*_ = **A**−**a**_*m*_

***Procedure***

        •          *Initialize the residual* **r**_0_ = **A**

        •          *Solve a least square problem to obtain a new vector* **x**_*t*_ = arg min_*x*_‖**Mx** − **A**‖

        •          *Find the new estimate*

                **a**_*t*_ = **M**_*t*_**x**_*t*_

                **r**_*t*_ = **A** − **a**_*t*_

                $\hat{w}=x_{j}$

                min ‖**w**‖_1_          *s*.*t*.          ‖**A**−**Mw**‖ < *ε*

where *ε* is the error. In Eq (1) we look to seek thereduced number of non-zero weights excitation $\hat{w}$. Matching with the existing techniques resolved with *L*_2_ minimization, this paper performs the array correction problem as greedy sparse optimization and convert the *L*_2_ norm to *L*_1_ norms. The *L*_2_ norm is computationally expensive and requires a large time to get the desired pattern. On the other hand, the *L*_1_ norm is convex and promotes sparsity in the solution and is computationally efficient. Moreover the *L*_1_ minimization gets the wanted pattern with the reduced number of sensors.

## Simulation results

In this section, simulation results are offered to confirm the efficiency of the proposed (GSCO) technique for failure correction in array antennas.

### Correction of failure with Chebyshev array

In this case, a Chebyshev (test array) uniform linear array of 20 numbers of sensors is considered to check the validity of the proposed GSCO technique for failure correction. The sidelobes of the test array is taken as -35 dB and Table 2 shows the excitation weights for the test array antenna. The Chebyshev radiation power pattern of these weights is depicted in Fig 3 by the black solid lines. The damage of sensors in the antenna array disturbs the entire pattern. Due to this damage, the communication becomes a dream. To get the original pattern back with the minimum number of array sensors is important, especially in radar and satellite communications. The damage sensor weight is represented by zero amplitude as given in Table 2. Fig 3 shows the radiation pattern of the Chebyshev, faulty and the pattern recovered by proposing greedy sparseness constrained optimization (GSCO) technique. From the simulation result, it is obvious that by the proposed technique, we received nearly the same pattern as the Chebyshev pattern. In this simulation, the mean square error (MSE) is used as the difference between the desired Chebyshev pattern and the estimated pattern obtained by the proposed technique. In this scenario, we assumed that four sensors (*w*_11_,*w*_12_,*w*_13_,*w*_14_) are damaged in an array of 20 sensors, i.e. 20% sensors are damaged. Due to this failure one cannot communicate. From Fig 3 it is clear that we get nearly the desired pattern from 16 numbers of sensors by the proposed method. So, the proposed method is very effective in case of failure and one can get the desired pattern with minimum number of sensors. The weights of the recovered pattern obtained by the proposed method are given in Table 2. The proposed method recovers the desired pattern in terms of sidelobes, null depth level and main beam width nearly the same as that of the original Chebyshev array. The MSE in this case is 2.1e-3 while the computation time for the recovery of the desired pattern is 2.7 s and require 97 number of samples to get the desired pattern. The values of sidelobes level, NDL and nulls are palced at the desired angles by the proposed GSCO as depicted in Table 3. In the second case, we consider the Chebyshev array of 32 numbers of sensors, but this time, consider the failure of six sensors *w*_17_,*w*_18_,*w*_19_,*w*_20_,*w*_21_,*w*_22_, due to which radiation pattern disturbs badly. From the results of Fig 4, it is clear that the desired pattern is recovered by the proposed method from 26 numbers of sensors. In this case, the MSE is 2.7e-2. For the recovery of the desired pattern, the proposed method require 105 number of samples. From the simulation results it is clear that if the array size increases the MSE also increases. The computation time required to recover the pattern is 3.4 s. The weight and positions of the recovered pattern obtained by the proposed GSCO are given in Table 4.

**Fig 3:**
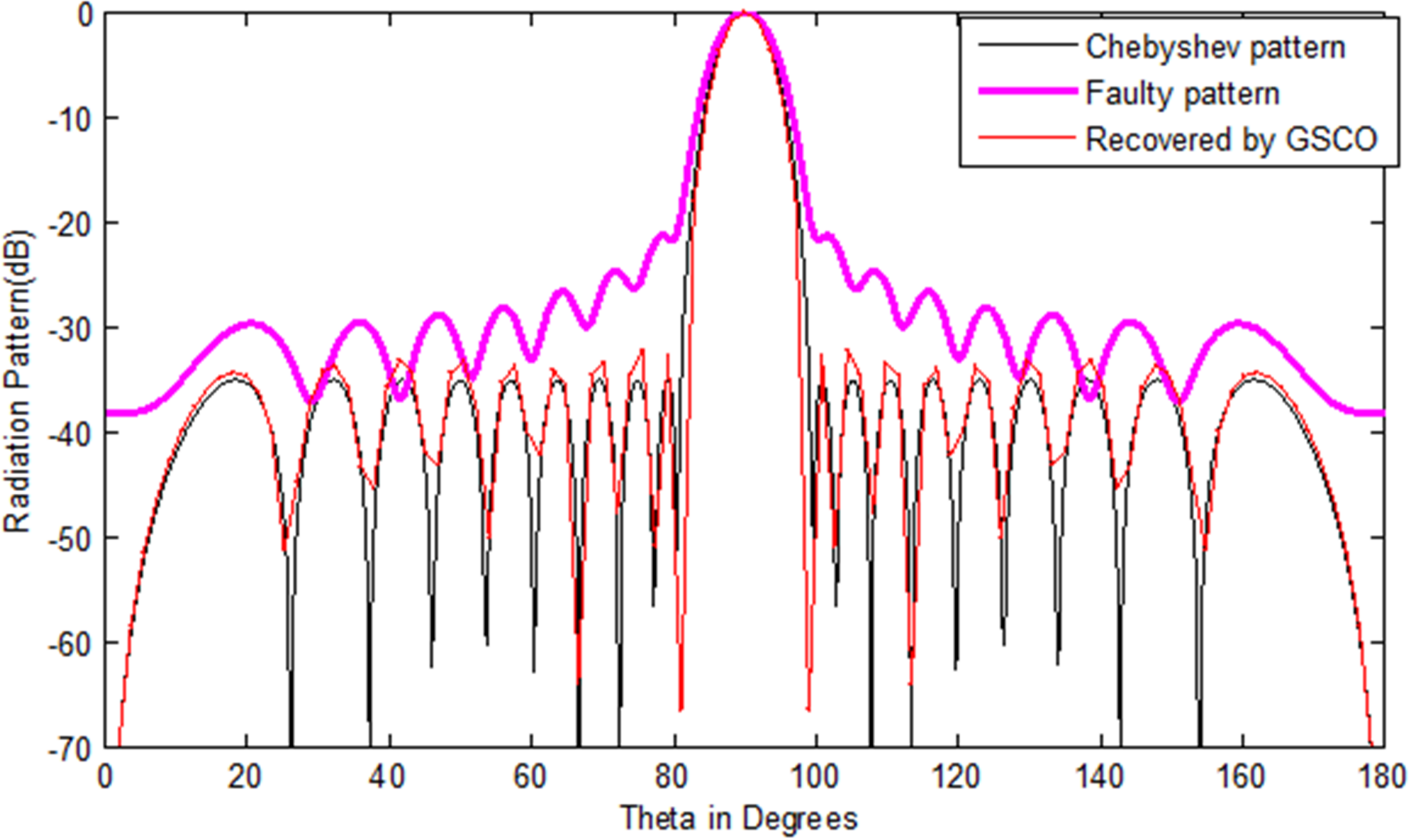
Chebyshev pattern of 20 numbers of sensors recovered by GSCO technique.

**Fig 4:**
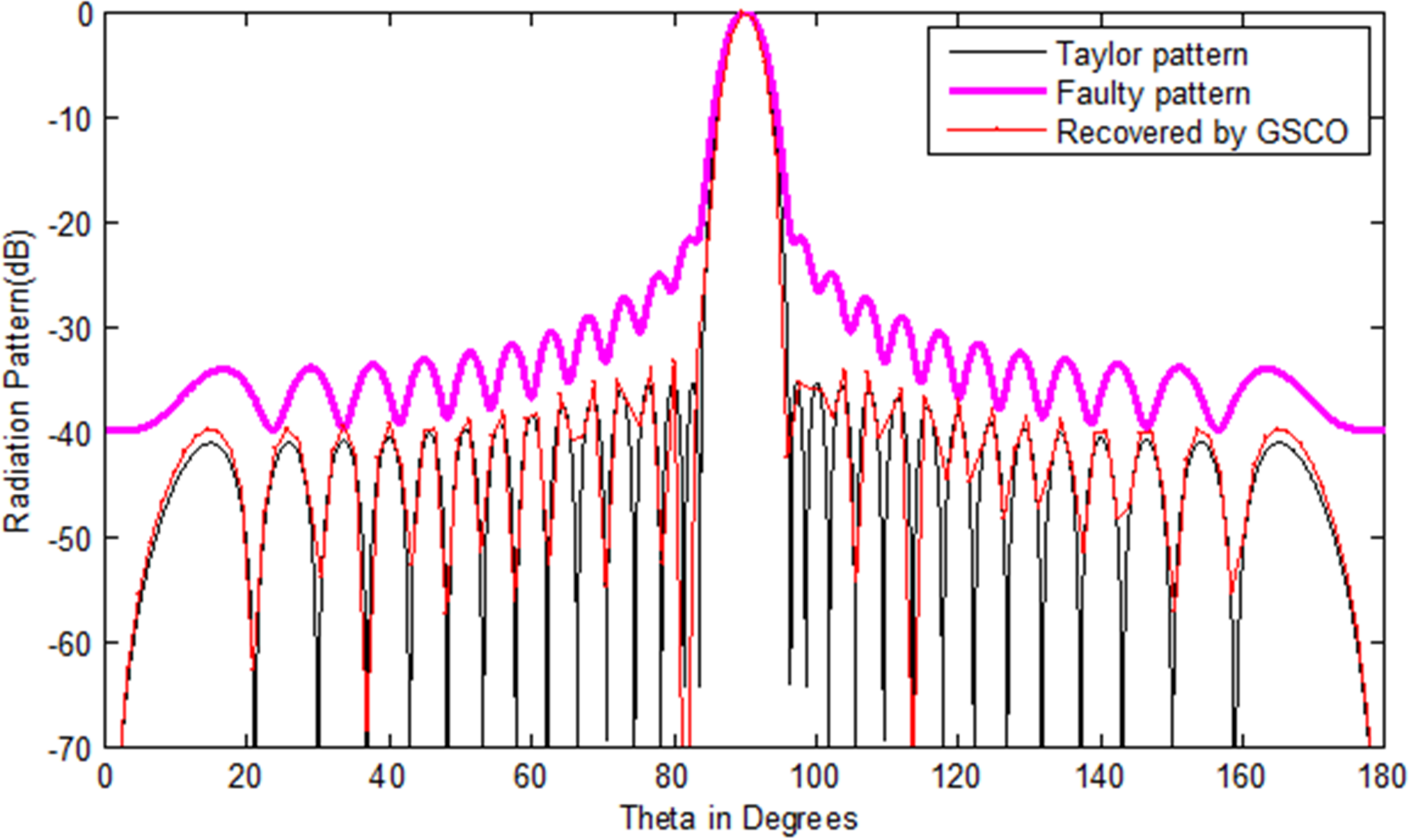
Chebyshev pattern of 32 numbers of sensors recovered by GSCO technique.

**Table 2:** Excitation weights of Chebyshev, faulty and recovered pattern.

Element No	Chebyshev weights N = 20	Faulty weights	Recovered by GSCO	
S/No	*a*_*i*_	*a*_*dam*_	*d*_*i*_/*λ*	*w*_*i*_
1	1.0000	1.0000	0.50	1.00
2	0.9644	0.9644	1.31	0.93
3	0.8962	0.8962	2.12	0.81
4	0.8013	0.8013	2.93	0.64
5	0.6875	0.6875	3.74	0.45
6	0.5636	0.5636	4.55	0.31
7	0.4389	0.4389	5.36	0.26
8	0.3215	0.3215	6.17	0.09
9	0.2180	0.2180	6.17	0.09
10	0.1934	0.1934	5.36	0.26
11	0.1934	0.0000	4.55	0.31
12	0.2180	0.0000	3.74	0.45
13	0.3215	0.0000	2.93	0.64
14	0.4389	0.0000	2.12	0.81
15	0.5636	0.5636	1.31	0.93
16	0.6875	0.6875	0.50	1.00
17	0.8013	0.8013		
18	0.8962	0.8962		
19	0.9644	0.9644		
20	1.0000	1.0000		

**Table 3:** Comparison analysis for initial, damaged and recovered array by GSCO.

Initial array Parameter			Damaged array parameter			Recovered by GSCO	
Null positions at an angle *θ*	SLL (dB)	NDL (dB)	Null positions	SLL (dB)	NDL (dB)	SLL (dB)	NDL (dB)
2.1	-35.00	-70.00	-37.17	-29.69	-37.03	-34.28	-70
25.8	-35.00	-70.00	-36.83	-29.52	-36.48	-33.72	-51.17
36.9	-35.00	-70.00	-33.21	-28.87	-33.99	-33.51	-45.28
45.9	-35.00	-62.21	-32.86	-28.17	-32.63	-33.51	-43.14
53.7	-35.00	-60.35	-29.98	-26.62	-29.78	-33.11	-50.02
60.3	-35.00	-62.76	-26.32	-24.71	-26.31	-33.72	-43.28
66.9	-35.00	-70.00	-21.53	-21.29	-21.08	-35.54	-63.98
72.6	-35.00	-70.00				-34.59	-50.34
77.1	-35.00	-56.57				-32.69	-50.85

**Table 4:** Excitation weights of Chebyshev, faulty and recovered pattern.

Element No	Chebyshev weights N = 32	Faulty weights	Recovered by GSCO technique	
S/No	*a*_*i*_	*a*_*dam*_	*d*_1_/*λ*	*w*_*i*_
1	1.0000	1.0000	0.45	1.0000
*2*	0.9863	0.9863	1.31	0.9714
*3*	0.9594	0.9594	2.12	0.9082
*4*	0.9202	0.9202	2.93	0.8375
*5*	0.8700	0.8700	3.74	0.7106
6	0.8103	0.8103	4.55	0.5810
7	0.7431	0.7431	5.36	0.4548
*8*	0.6703	0.6703	6.17	0.3610
*9*	0.5943	0.5943	6.61	0.2187
10	0.5170	0.5170	7.21	0.1971
11	0.4406	0.4406	7.73	0.1131
12	0.3669	0.3669	8.31	0.091
13	0.2976	0.2976	8.90	0.004
4	0.2341	0.2341	8.90	0.004
15	0.1774	0.1774	8.31	0.091
16	0.2503	0.2503	7.73	0.1131
17	0.2503	**0. 0000**	7.21	0.1971
18	0.1774	**0.0000**	6.61	0.2187
19	0.2341	**0.0000**	6.17	0.3610
20	0.2976	**0.0000**	5.36	0.4548
21	0.3669	**0.0000**	4.55	0.5810
22	0.4406	**0.0000**	3.74	0.7106
23	0.5170	0.5170	2.93	0.8375
24	0.5943	0.5943	2.12	0.9082
25	0.6703	0.6703	1.31	0.9714
26	0.7431	0.7431	0.45	1.0000
27	0.8103	0.8103		
28	0.8700	0.8700		
29	0.9202	0.9202		
30	0.9594	0.9594		
31	0.9863	0.9863		
32	1.0000	1.0000		

### Correction of failure with Taylor pattern

In this example, a Taylor array of 30 numbers of sensors with sidelobes level -35 dB is taken as the test array. We assumed that six numbers of sensors *w*_16_,*w*_17_,*w*_8_,*w*_9_,*w*_20_,*w*_21_ get damaged in the array. Due to which the pattern disturbs severely. Its sidelobes level increases and nulls are damaged. In such critical situation, the communications become a dream. Now the main job is to recover the wanted pattern with reduced number of sensors by adjusting the weights and distance in the antenna array. To check the validity of the proposed method, we assume the failure of six sensors in an array of 30 sensors, i.e. 20% sensors are damaged. As one can see in Fig 5, due to this failure the whole pattern disturbs. The require is to get the desired pattern from 24 numbers of sensors by the proposed GSCO technique. Table 5 shows the weights of the Taylor pattern, damaged pattern and the weights and positions of the recovered pattern by the proposed technique. The red dotted line in Fig 5 shows the pattern obtained by the proposed GSCO technique. The MSE between the wanted and the estimated radiation pattern is 3.02e-2. The recovered pattern is obtained from the 24 number of sensors by the proposed method which require 20% less number of sensors and get the same pattern as that of the original Taylor array.The same scenario of 30 number of sensors is taken for Chebyshev array with sidelobes level -35 dB. Again we consider the six number of failures as that in Taylor array. From Fig 6, it is clear that due to six sensor failure, the pattern disturbs badly in terms of sidelobes level, null depth level and nulls are shifted from their original positions. By applying the proposed GSCO method, the desired pattern is recovered from 24 number of sensors which is depicted in Fig 6.

**Fig 5:**
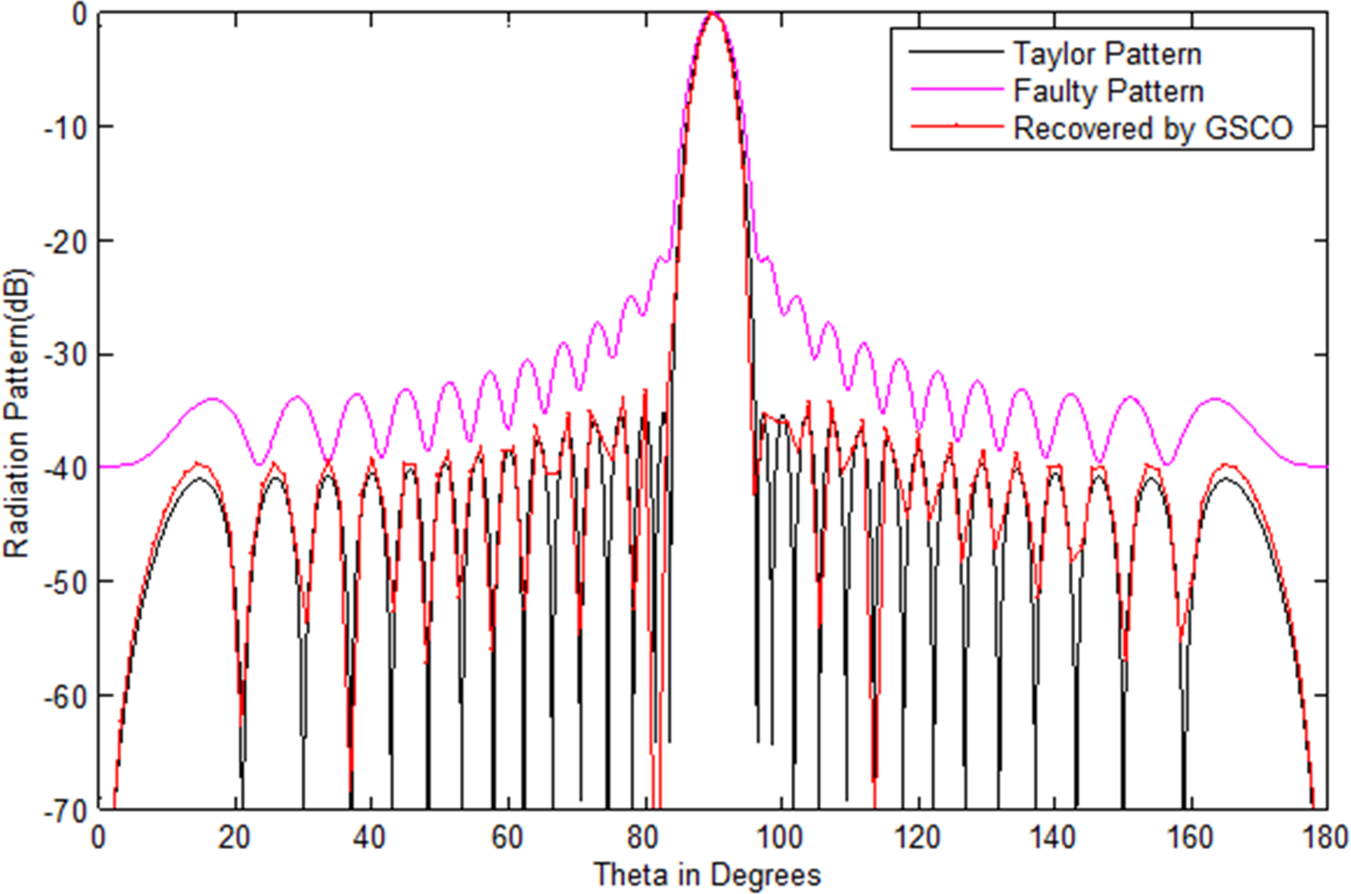
Taylor pattern of 30 numbers of sensors with sidelobes -35 dB recovered by GSCO technique.

**Fig 6:**
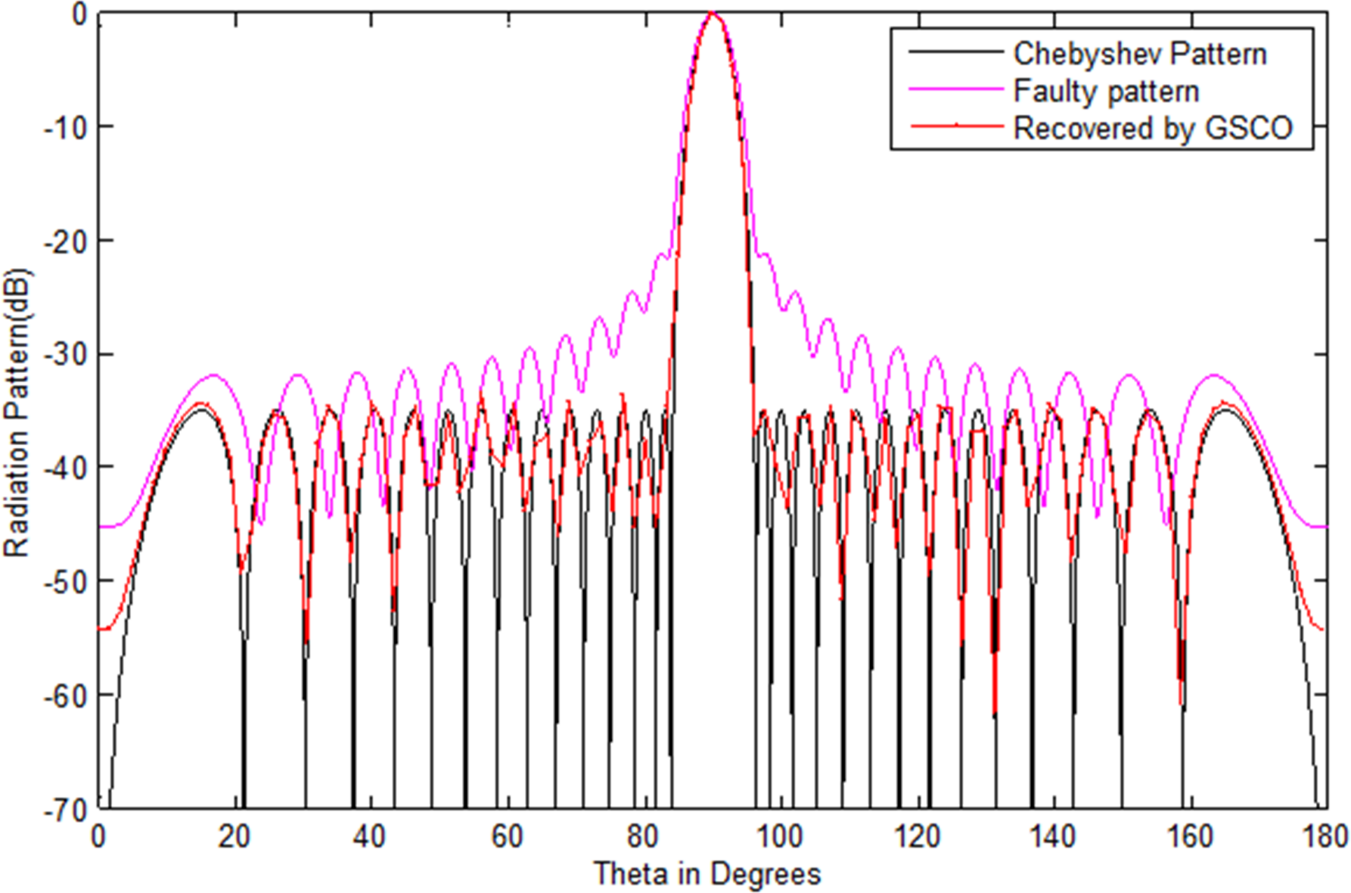
Chebyshev pattern of 30 numbers of sensors with sidelobes -35 dB recovered by GSCO technique.

**Table 5:** Excitation weights of Taylor pattern, faulty and recovered pattern.

Element No	Taylor array weights N = 30	Faulty weights	Recovered by GSCO	
S/No	*a*_*i*_	*a*_*dam*_	*d*_*i*_/*λ*	*w*_*i*_
1	1.0000	1.0000	0.53	1.000
*2*	0.9844	0.9844	1.34	0.9243
*3*	0.9538	0.9538	2.15	0.8521
*4*	0.9094	0.9094	2.95	0.8173
*5*	0.8527	0.8527	3.75	0.7482
6	0.7860	0.7860	4.55	0.6585
7	0.7115	0.7115	5.35	0.5763
*8*	0.6318	0.6318	6.15	0.4887
*9*	0.5495	0.5495	6.95	0.3641
10	0.4673	0.4673	7.75	0.2019
11	0.3874	0.3874	8.55	0.1985
12	0.3121	0.3121	9.35	0.1021
13	0.2430	0.2430	9.35	0.1021
4	0.1815	0.1815	8.55	0.1985
15	0.2402	0.2402	7.75	0.2019
16	0.2402	**0.0000**	6.95	0.3641
17	0.1815	**0.0000**	6.15	0.4887
18	0.2430	**0.0000**	5.35	0.5763
19	0.3121	**0.0000**	4.55	0.6585
20	0.3874	**0.0000**	3.75	0.7482
21	0.4673	**0.0000**	2.95	0.8173
22	0.5495	0.5495	2.15	0.8521
23	0.6318	0.6318	1.34	0.9243
24	0.7115	0.7115	0.53	1.000
25	0.7860	0.7860		
26	0.8527	0.8527		
27	0.9094	0.9094		
28	0.9538	0.9538		
29	0.9844	0.9844		
30	1.0000	1.0000		

### Correction of failure with large arrays

In order to check the validity of the proposed GSCO technique for large arrays usually used in satellite and radar communications systems. At the first instant, we consider a large linear array of 100 numbers of sensors of Chebyshev pattern with sidelobes level -40 dB as shown in Fig 7 and assumed that 10 number of sensors *w*_51_,*w*_52_,*w*_53_,*w*_54_,*w*_55_,*w*_56_,*w*_57_,*w*_58_,*w*_59_,*w*_60_ are getting damaged. As one can clearly observe that due to 10 sensor damage, the pattern get damaged badly. By applying the proposed method, the pattern can be recovered from 90 numbers of sensors. The recovered pattern shown in Fig 7 by the red dotted lines is approximately the same as that of the original Chebyshe array. The MSE for the recovered pattern is 4e-2. The time taken for the recovery of the desired is 9.3 s which is much less than the evolutionary computational techniques. The recovery of the desired pattern by the proposed technique in short time shows its effectiveness.

**Fig 7:**
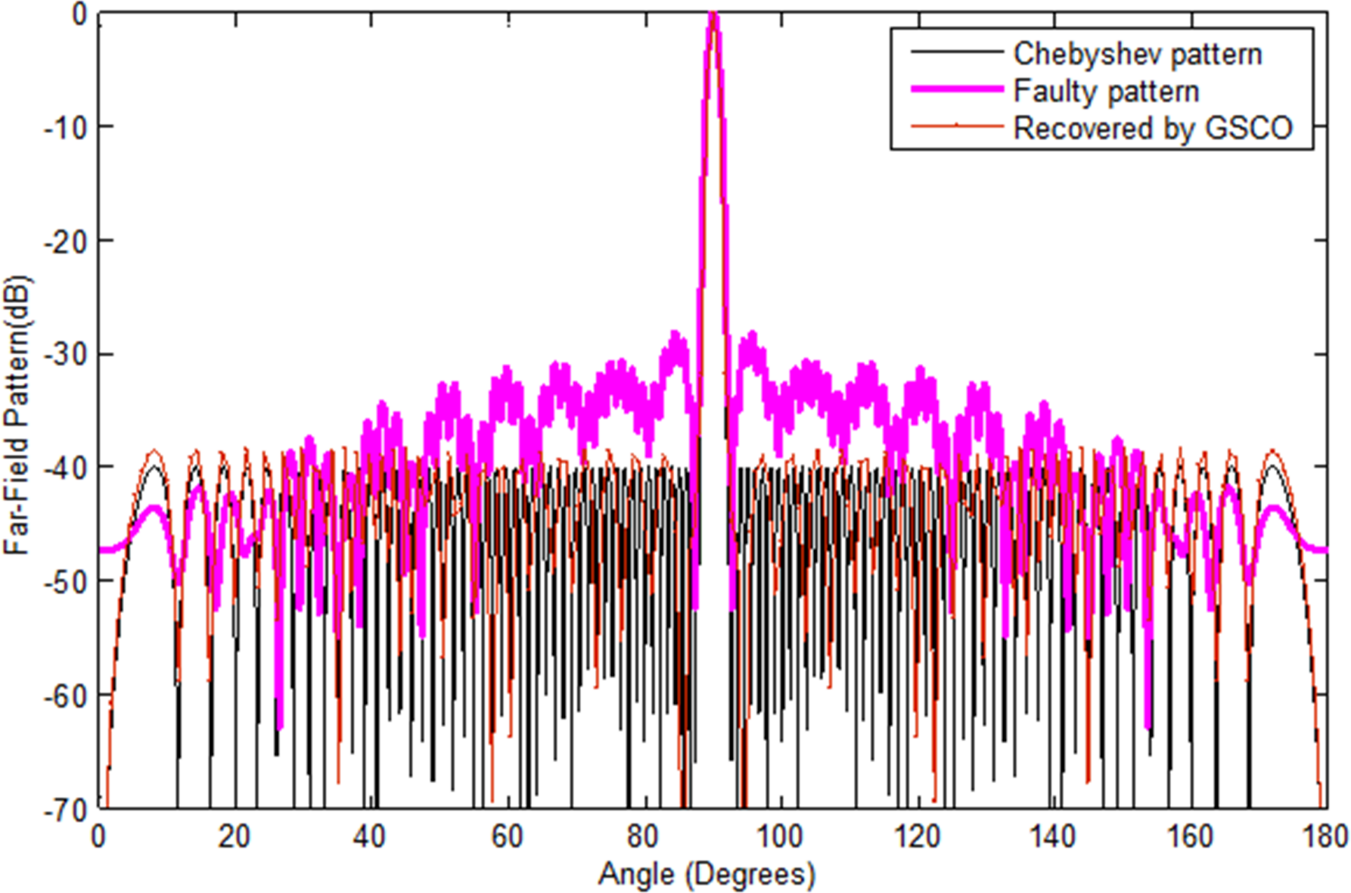
Chebyshev pattern of 100 sensors with sidelobes -40 dB recovered by GSCO technique.

In the second case, the Taylor pattern of 100 numbers of sensors with sidelobes level -40 dB is taken to check the validity of the proposed technique which is depicted in Fig 8. Now we consider the failure of 10 sensors *w*_51_,*w*_52_,*w*_53_,*w*_54_,*w*_55_,*w*_56_,*w*_57_,*w*_58_,*w*_60_,*w*_61_ at different positions. In antenna arrays, the position of failure is very important. If the sensors get damaged near the center of the array, then it disturbs the pattern badly as compared to the corner element failure. The proposed method recovered the pattern in terms of sidelobes, nulls and main beam width by adjusting the weights and positions of the remaining sensors in the array. The recovered pattern is shown in Fig 8 by the red dotted line. In Fig 9, we have assumed the failure of random number of sensors *w*_1_,*w*_3_,*w*_4_,*w*_7_. One can clearly monitor that due to random failure the Chebyshev power pattern disturbs badly. By applying the proposed method, the desired pattern is recovered which is depicted in Fig 9 by the red dotted lines.

**Fig 8:**
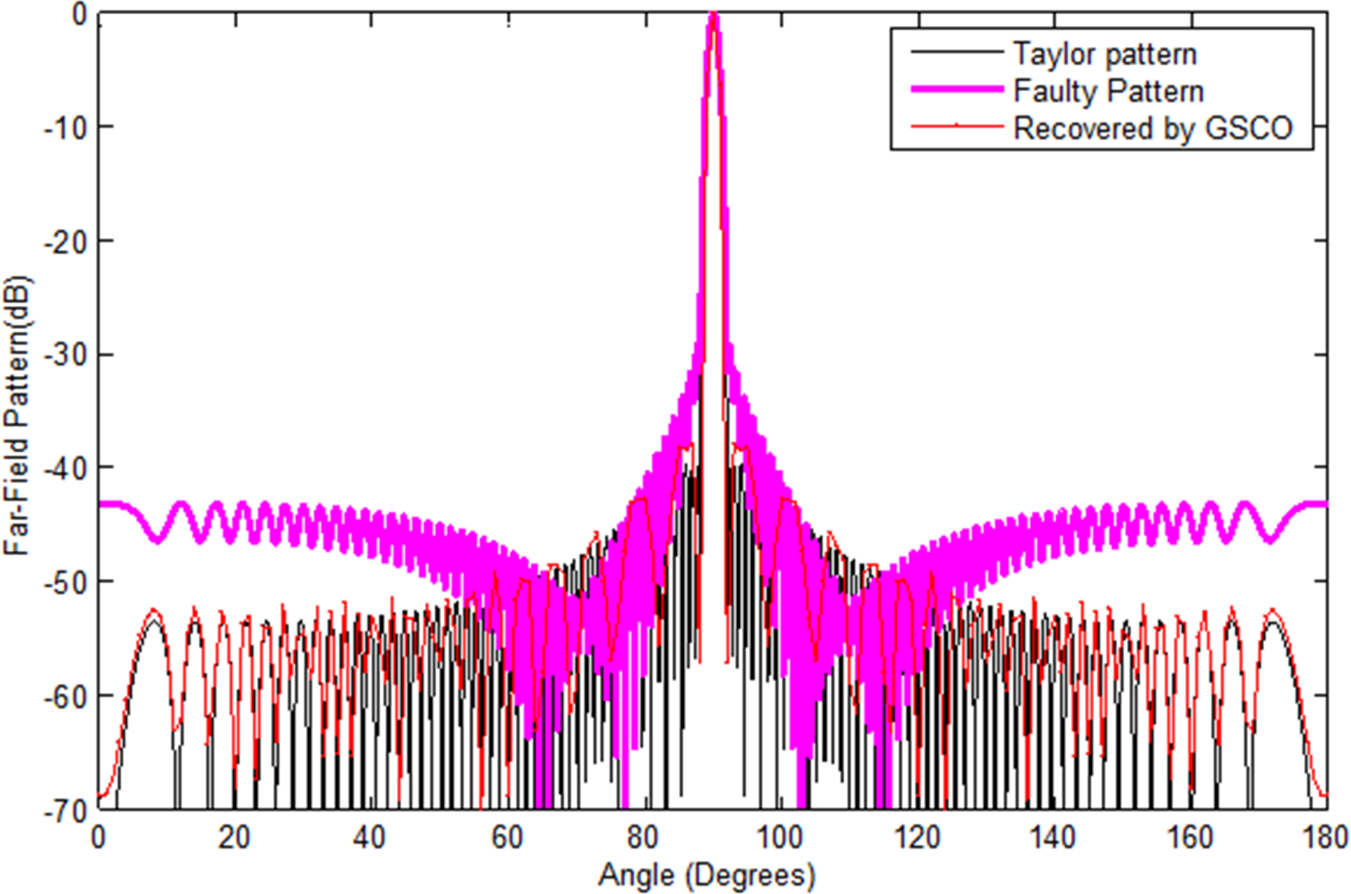
Taylor pattern of 100 sensors with sidelobes -40 dB and n = 4 recovered by GSCO technique.

**Fig 9:**
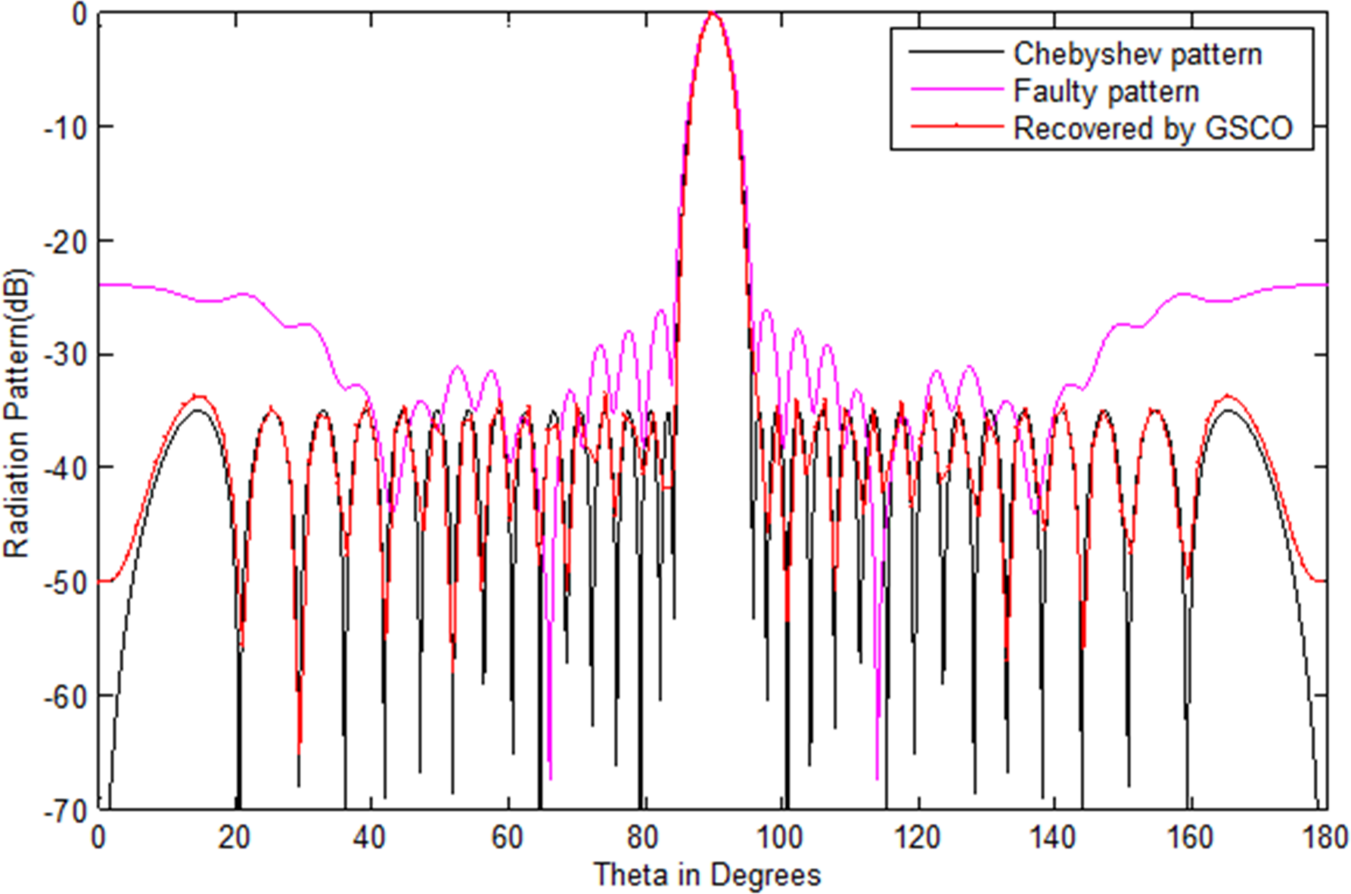
Chebyshev pattern of 32 number of sensors with random number of failure *w*_1_,*w*_3_,*w*_4_,*w*_7_ and sidelobes -40 dB recovered by GSCO technique.

### Comparison with the existing techniques

The proposed technique, performance is compared with the existing technique [25–26]. The performance parameter for comparison is sidelobes level, null depth level, number of nulls and the computational time. The proposed method recovered the desired pattern with minimum number of sensors as compared to the existing techniques. In [25], sidelobes are recovered only by adjusting the remaining number of sensors while [26] recovers the sidelobes and some null but not at the required positions. Moreover, it requires more computation to get the desired pattern. On the other hand, our proposed technique recovers the sidelobes, nulls at their desired locations and require less computation time. The comparative analysis of existing and proposed technique are given in Table 6. By the proposed technique, we get the desired power pattern in terms of sidelobes, number of nulls and null depth level with minimum number of sensors. In Fig 10, we have compared the proposed method with the conventional method [25]. In this case assumed the failure of (*w*_2_,*w*_5_,*w*_6_) sensors in an array of 32 number of sensors. The convention method recovers the sidelobe level but can not solve the issues of null placement at the desired locations. But our proposed method recovered the sidelobe level and null placement at the desired locations as shown in Fig 10. We can steer the main beam direction if the desired user changes their direction. In this case the main beam is poininting in the direction of wanted user at an angle of 120 degeree along the direction of nulls at the desired locations as depicted in Fig 11.

**Fig 10:**
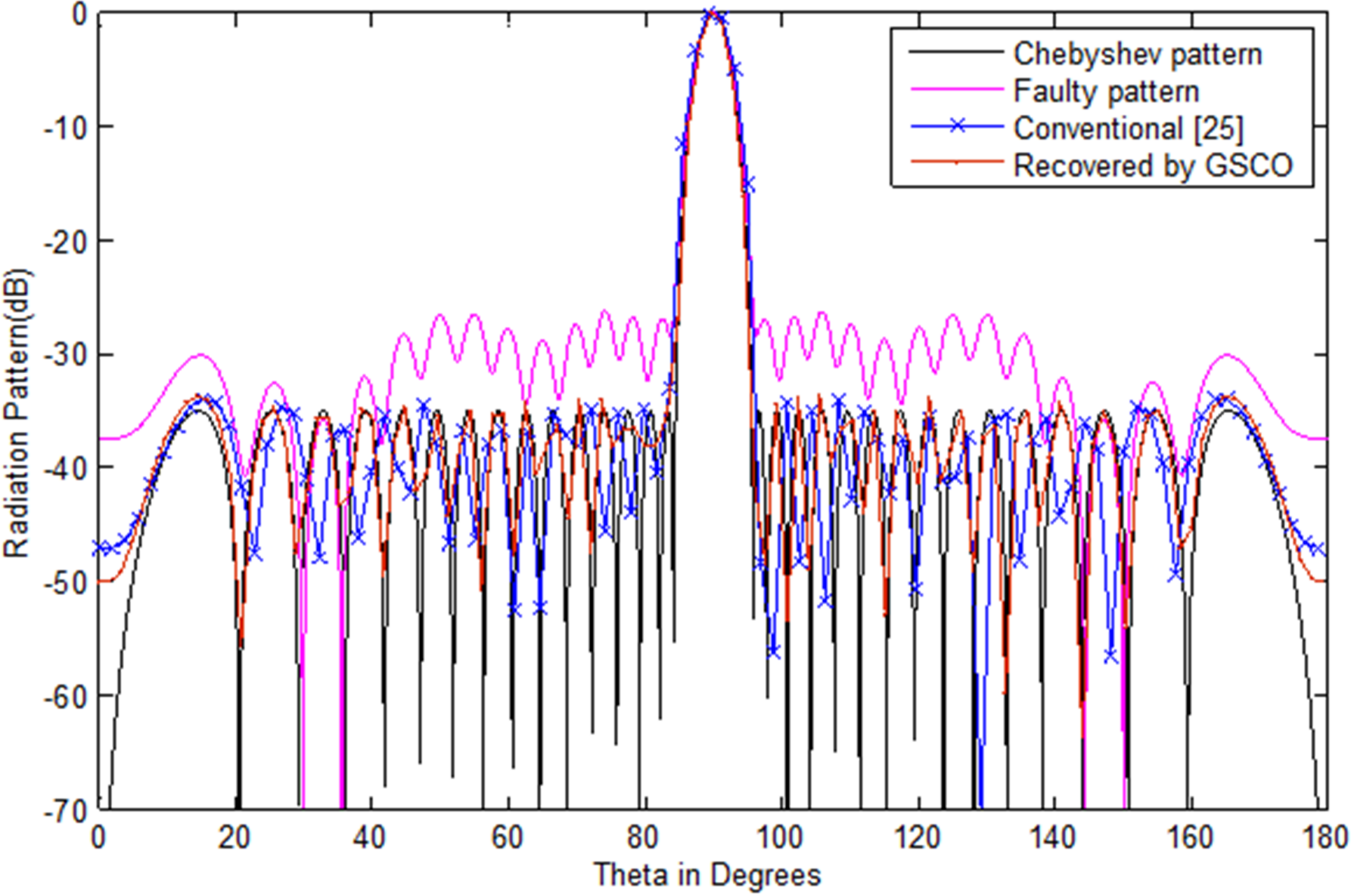
Chebyshev pattern of the conventional [25] and proposed method with random number of failure (*w*_2_,*w*_5_,*w*_6_).

**Fig 11:**
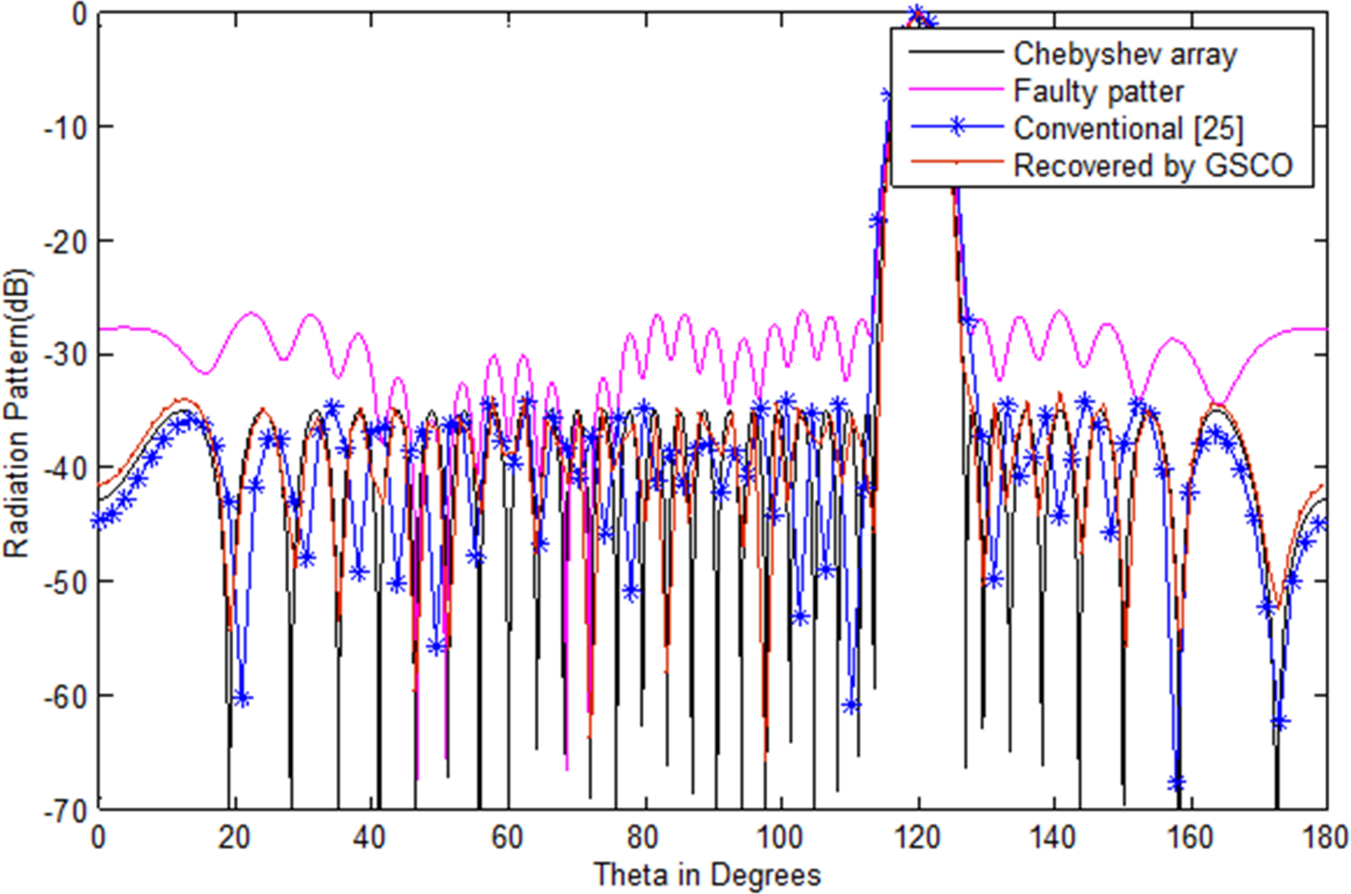
Chebyshev pattern of the conventional [25] and proposed method with random number of failure (*w*_2_,*w*_5_,*w*_6_) and main beam pointing at an angle *θ* = 120°.

**Table 6:** Comparison with the existing techniques.

S/No	Parameters of Pattern	Proposed method	Conventional method [25]	Conventional method [26]
1	Number of sensor	32	32	32
2	Sidelobes level	-34 dB	-34 dB	-30 dB
3	Null depth level	-60 dB	-45 dB	-45 dB
4	Number of nulls recovered	29	6	14
5	Number of faulty sensors	6	3	2
6	Time	3.4 sec	NA	NA
7	MSE	2.7e-2	0.4	NA
8	Number of samples	105	NA	NA

In this case, we have compared the error analysis and convergence rate analysis for different number of sensors by the proposed greedy method and conventional method which is shown in Figs 12 and 13. Fig 13 shows the error versus minimum number of sensors for the recovery of the desired power pattern by the conventional [20] and proposed method. Our proposed method recovers the desired pattern with reduced number of sensors as compared to conventional genetic algorithm [20]. The greedy algorithms require relatively less effort as compared to evolutionary algorithms such as genetic algorithm etc. in terms of error and convergence rate analysis. The estimate is reliable in terms of sidelobes level, null depth level and nulls recovery of the desired pattern. Fig 13 shows the error analysis by the proposed GSCO and conventional method. As can be seen from Fig 11, the proposed GSCO recovers the desired pattern with reduced number of sensors in terms of sidelobes level, null depth level and placement of nulls at the desired locations.

**Fig 12:**
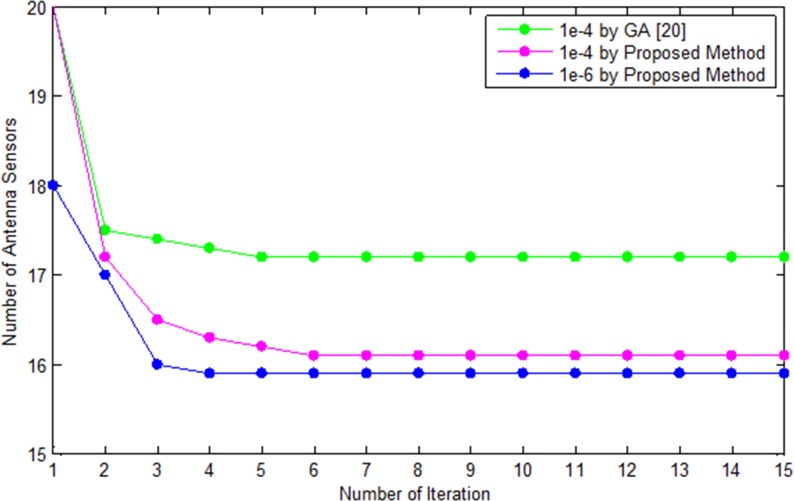
Convengence of the conventional [20] and proposed method at different values of errors.

**Fig 13:**
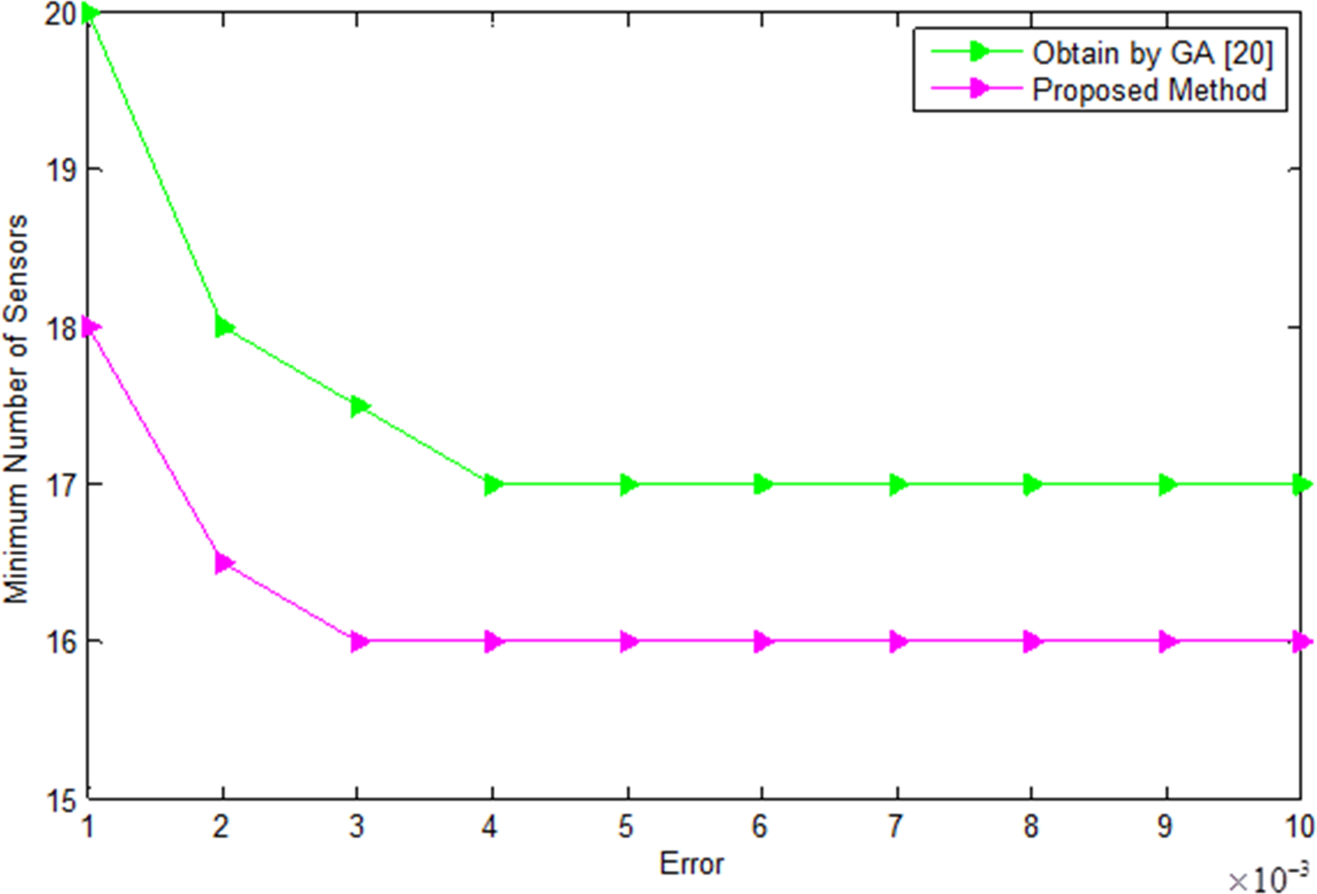
Error versus minimum number of sensors by conventional [20] and Proposed method.

## Conclusion

In this paper, the array antenna failure correction problem is developed from the greedy sparseness constrained optimization (GSCO) technique. The available failure correction techniques are based on the readjustment of the active sensors in the array antenna to recover the desired pattern. But the proposed technique taking the advantage of sparseness in terms of sensor location and the failure correction problem is ensemble as an optimization problem and solved by GSCO technique. The pattern recovered by the proposed technique has desired sidelobes level and number of nulls require less simulation time as compared to the existing techniques. Simulation results are offered to show the effectiveness of linear array failure correction problem with GSCO. This method can be extended to circular arrays.

## Supporting information

S1 File Code. (DOCX)
