# Supplementary material for: Correction of failure in linear antenna arrays with greedy sparseness constrained optimization technique
Source: PLoS One. 2017 Dec 18;12(12):e0189240. doi: 10.1371/journal.pone.0189240 (PMC5734759; doi:10.1371/journal.pone.0189240)
Supplement: S1 File — (DOCX) [file pone.0189240.s001.docx]

Code

clc;

clear all;

home;

lambda=1;

d=lambda/2;

k=2*pi/lambda;

pts = 40;

theta= 0:0.3:180;

u = (pi*d/lambda)*(cosd(theta)-cosd(90));

u=u';

w=[1.0000 0.9644 0.8962 0.8013 0.6875 0.5636 0.4389 0.3215 0.2180 0.1934 0.1934 0.2180 0.3215 0.4389 0.5636 0.6875 0.8013 0.8962 0.9644 1.0000];

v = [exp(j*u), exp(3j*u), exp(5j*u), exp(7j*u), exp(9j*u),exp(11j*u), exp(13j*u), exp(15j*u), exp(17j*u), exp(19j*u), exp(-19j*u), exp(-17j*u), exp(-15j*u) , exp(-13j*u), exp(-11j*u), exp(-9j*u), exp(-7j*u), exp(-5j*u) , exp(-3j*u), exp(-j*u)];

p = (v)*(w');

p=20*log10(p/max(p)); %normalized power in db

plot (theta,(p),'k');

axis([0, 180,-70,0])

xlabel ('Theta in Degrees');

ylabel ('Radiation Pattern(dB)');

hold on;

%%%%%%%%%%%%%%%%%%%%%%%%%%%%%% w4 are faulty%

w=[1.0000 0.9644 0.88962 0.8013 0.6875 0.5636 0.4389 0.3215 0.2180 0.1934 0.00000001934 0.0000002180 0.000003215 0.0000004389 0.5636 0.6875 0.8013 0.8962 0.9644 1.0000];

v = [exp(j*u), exp(3j*u), exp(5j*u), exp(7j*u), exp(9j*u),exp(11j*u), exp(13j*u), exp(15j*u), exp(17j*u), exp(19j*u), exp(-19j*u), exp(-17j*u), exp(-15j*u) , exp(-13j*u), exp(-11j*u), exp(-9j*u), exp(-7j*u), exp(-5j*u) , exp(-3j*u), exp(-j*u)];

p = (v)*(w');

p=20*log10(p/max(p)); %normalized power in db

plot (theta,(p),'r');

%%%%%%%%%%%%%%%%%%%%%%%%%%%%% Recovered%%%%%%%% %%%%%%%%%%%%%%%%%%%

lambda=1;

d=lambda/2;

k=2*pi/lambda;

pts = 40;

theta= 0:1.8:180;

u1 = (pi*d/lambda)*(cosd(theta)-cosd(90));

u1=u1';

wrec=Fucntion[];

vrec = [exp(j*u1), exp(3j*u1), exp(5j*u1), exp(7j*u1), exp(9j*u1),exp(11j*u1), exp(13j*u1), exp(15j*u1), exp(17j*u1), exp(19j*u1),exp(-11j*u1), exp(-9j*u1), exp(-7j*u1), exp(-5j*u1) , exp(-3j*u1), exp(-j*u1)];

p = (vrec)*(wrec');

p=20*log10(p/max(p)); %normalized power in db

plot (theta,(p),'b');
